# A Novel Model for Identifying Essential Proteins Based on Key Target Convergence Sets

**DOI:** 10.3389/fgene.2021.721486

**Published:** 2021-07-29

**Authors:** Jiaxin Peng, Linai Kuang, Zhen Zhang, Yihong Tan, Zhiping Chen, Lei Wang

**Affiliations:** ^1^College of Computer, Xiangtan University, Xiangtan, China; ^2^College of Computer Engineering and Applied Mathematics, Changsha University, Changsha, China

**Keywords:** protein-protein interaction, essential protein, heterogeneous network, random walk with restart, key target convergence set

## Abstract

In recent years, many computational models have been designed to detect essential proteins based on protein-protein interaction (PPI) networks. However, due to the incompleteness of PPI networks, the prediction accuracy of these models is still not satisfactory. In this manuscript, a novel key target convergence sets based prediction model (KTCSPM) is proposed to identify essential proteins. In KTCSPM, a weighted PPI network and a weighted (Domain-Domain Interaction) network are constructed first based on known PPIs and PDIs downloaded from benchmark databases. And then, by integrating these two kinds of networks, a novel weighted PDI network is built. Next, through assigning a unique key target convergence set (KTCS) for each node in the weighted PDI network, an improved method based on the random walk with restart is designed to identify essential proteins. Finally, in order to evaluate the predictive effects of KTCSPM, it is compared with 12 competitive state-of-the-art models, and experimental results show that KTCSPM can achieve better prediction accuracy. Considering the satisfactory predictive performance achieved by KTCSPM, it indicates that KTCSPM might be a good supplement to the future research on prediction of essential proteins.

## Introduction

With the deepening of researches on proteins, accumulating evidences have demonstrated that proteins are closely related to most of the life activities. Moreover, different proteins are of different importance to different life activities. Among these proteins, essential proteins, as a kind of important proteins, are essential for the survival, and development of life. Therefore, in recent years, detection and recognition of essential proteins has become a hot issue in the research and development of disease treatment. However, it is very time-consuming and expensive to identify essential proteins by traditional biological experiments, which leads to the emergence and development of computational prediction methods. For instance, Zhao et al. (2019) designed a new random walk wandering based prediction model to detect key proteins based on a heterogeneous network consisting of proteins and protein domains. Jeong et al. (2001) found that PPI networks are scale-free and proposed a center-lethal rule for PPI networks. Based on which, lots of methods including the information centrality (IC) (Stephenson and Zelen, 1989), betweenness centrality (BC) (Joy et al., 2014), degree centrality (DC) (Hahn and Kern, 2004), Closeness Centrality (CC) (Wuchty and Stadler, 2003), subgraph centrality (SC) (Estrada and Rodriguez-Velazquez, 2005), and neighbor centrality (NC) (Wang et al., 2012) had been put forward successively. In addition, Yu et al. (2007) proposed the importance of network bottlenecks. Estrada (2006) found that a small number of binary proteins were mostly essential proteins. Chua et al. (2006) proposed to identify essential proteins by calculating the weights of indirect neighbor nodes. Li et al. (2012) designed a method to predict essential proteins by combining PPI networks with gene expression data of proteins. Li et al. (2011) find essential proteins by analyzing the relationship between proteins and their neighbors, and define the method as LAC. Peng et al. (2012) combined orthology information of proteins with PPI networks to predict key proteins. Zhao et al. (2014) found that combination of gene expression profiles and PPI networks was of great help to the prediction accuracy of essential proteins. Min et al. (2017) discovered that the complex information of proteins can improve prediction accuracy and precision of potential essential proteins. Zhao et al. (2016) proposed a basic protein identification method based on protein gene time expressions and protein domains. Zhang et al. (2019) proposed a new protein prediction method called TEGS, which can identify essential proteins by fusing the introduced multiple biological information data. Lei et al. (2018) found that it can achieve good results to adopt artificial fish swarm optimization algorithm into key protein prediction. Peng et al. (2015) discovered that combination of protein domain features and protein interaction networks can effectively predict potential essential proteins. Li et al. (2019) proposed a target convergence set (TCS) based method for predicting potential lncRNA-disease associations. Athira and Gopakumar (2020) proposed a multiplex network to identifying essential proteins. Zhang et al. (2018) designed a novel method by combining network topology, gene expression profile and GO information to identifying essential proteins. Fan et al. (2017) proposed a modified PageRank algorithm based on subcellular information. Meng et al. (2021) predict the essential protein by constructing a new weighted protein and protein domain network, and performing a local random walk on this basis. Xenarios et al. (2002) introduced a public database called DIP for studying cellular networks of protein interactions. Gavin et al. (2006) provided a complete and comprehensive eukaryotic machine and biological data integration and modeling platform.

Inspired by above methods, in this manuscript, a computational model named KTCSPM was proposed to predict essential proteins. In KTCSPM, a weighted PDI network was first constructed by integrating a weighted PPI network and a weighted domain-domain interaction (DDI) network. And then, each node in the weighted PDI network would be assigned a unique key target convergence sets (KTCS) according to the network distance information of the weighted PDI network, which could reflect the specificity of different nodes in the process of random walk with restart and improve the predictive performance of KTCSPM. Next, for an arbitrarily selected walker, considering that there may still be some nodes that are essential proteins but not included in KTCS while KTCS reached the final convergence state, each node in the heterogeneous network would be further assigned a unique Intact Set (IS) to ensure that the predicted results would not be omitted as far as possible. Next, we will construct a random walk probability matrix and calculate the stable walk probability of all nodes, and then rank each protein based on the initial protein score vector. Finally, in order to evaluate the predictive performance of KTCSPM, we compared it with 12 advanced predictive methods based on two kinds of yeast PPI networks, and experimental results showed that KTCSPM can achieve reliable predictive accuracy of 90.19, 81.96, 70.72, 62.04, 55.83, and 51.13% in top 1, top 5, top 10, top 15, top 20, and top 25% of predicted key proteins separately, which are better than all these 12 competing predictive models.

## Materials and Methods

### Construction of the Weighted PPI Network

In this section, we will download known PPI data from two different public databases such as the DIP database (Xenarios et al., 2002) and the Gavin database (Gavin et al., 2006), respectively. Obviously, based on these known PPI network downloaded from any given public database, an original PPI network ***PPIN*** = < ***D_PP_***, ***E_PP_*** > can be constructed as follows: Let ***D_PP_*** = {***p_1_***, ***p_2_***, …, ***p_N_P__***} represent the set of newly downloaded proteins and ***E_PP_*** denote the set of edges between proteins in PPIN, here, for any two given proteins ***p_i_*** and ***p_j_*** in ***D_PP_***, if and only if there is a known interaction between them, then we define that there is an edge between them in PPIN. Thereafter, based on the newly constructed original PPI network PPIN, we can further obtain an ***N_P_*** × ***N_P_*** dimensional adjacency matrix ***M_PPIN_*** as follows: for any two given protein nodes ***p_i_*** and ***p_j_*** in PPIN, if and only if there is an edge between them in PPIN, there is ***M_PPIN_***(***p_i_***, **p_*j*_**) = 1, otherwise there is ***M_PPIN_***(***p_i_***, **p*_j_***) = 0.

In previous studies, the Gaussian interaction profile kernel similarity has been widely used to measure the similarity between similar nodes (Chen et al., 2016). In this section, for any two given proteins *p*_*i*_ and *p*_*j*_ in *M*_*PPIN*_, we define the Gaussian interaction profile kernel similarity between them as follows:


(1) $$\text{GKS}\left(i,j\right)=\text{exp}\left(-\gamma_{p}{||\text{IP}\left({\text{p}}_{i}\right)-\text{IP}\left({\text{p}}_{j}\right)||}^{2}\right)$$

(2) $$\gamma_{p}=\gamma_{p}^{\prime }/\sum_{k}^{N_{P}}=1{||\text{IP}\left({\text{p}}_{k}\right)||}^{2}$$

Here, *IP*(*p*_*t*_) represents the vector of elements in the *t-th* row of the matrix M_PPIN_, and γ_*p*_ denotes the normalized kernel bandwidth based on the bandwidth parameter $\gamma_{p}^{\prime }$. In addition, according to the methodology proposed by Vanunu et al. (2010), we will further optimize above Gaussian interaction profile kernel similarity of protein by introducing a logistics function as follows:


(3) $$LGKS\left(p_{i},p_{j}\right)=\frac{1}{1+e^{\left(-12GKS\left(i,j\right)+log9999\right)}}$$

This logistic function can make the calculated results of Gaussian interaction porofile kernel similarity more influential in the identification of essential proteins. Additionally, considering that while analyzing the topology structure of PPI network, the PPI network can be weighted to show the interaction between proteins, therefore, based on above newly obtained matrixLGKS, for any two given proteins *p_i_* and *p*_*j*_, we can weigh the relationship between them as follows:


(4) $$W_{PP}\left(p_{i},p_{j}\right)=\frac{LGKS\left(p_{i},p_{j}\right)+\frac{{\left|N\left(p_{i}\right)\cap N\left(p_{j}\right)\right|}^{2}}{\left(\left|N\left(p_{i}\right)+1\right|\right)*\left(\left|N\left(p_{j}\right)+1\right|\right)}}{2}$$

Here, *N*(*p*_*i*_)and *N*(*p*_*j*_) represent the sets of protein nodes directly adjacent to *p_i_* and *p_j_* in PPIN, respectively, and *N*(*p*_*i*_)∩*N*(*p*_*j*_) denotes the set of protein nodes adjacent to both *p_i_* and *p_j_* in PPIN. Obviously, based on above Equation (4), we can obtain a weighted PPI network *WPIN = < D*_*PP*_,*E*_*WPP*_> easily by taking *W*_*PP*_(*p*_*i*_, *p_j_*) as the weight of the edge between nodes *p_i_* and *p_j_* in WPIN, where *D*_*PP*_ and *E*_*WPP*_ denote the sets of nodes and edges in WPIN separately.

### Construction of the Weighted DDI Network

In this section, we will first download known domain data from the Pfam database (Peng et al., 2012; Bateman et al., 2014), and for convenience, let ***D_DD_*** = {***d_1_***, ***d_2_***, …, ***d**_**N_D_**_*} represent the set of newly downloaded domains, then for any given protein ***p_i_*** ∈ ***D_PP_***, and domain ***d_j_*** ∈ ***D_DD_***, it is obvious that we can estimate the relationship between them as follows:


(5) $${\text{W}}_{\mathrm{PD}}\left({\text{p}}_{i},{\text{d}}_{j}\right)=\frac{\sum_{p_{k}\in d_{j}}{\text{W}}_{\mathrm{PP}}\left({\text{p}}_{i},{\text{p}}_{k}\right)}{\left|{\text{d}}_{j}\right|}$$

Here, |***d_j_***| represents the number of different proteins belonging to ***d_j_***. Furthermore, according to above Equation (5), for any two given domains ***d_i_*** and ***d_j_*** in ***D_DD_***, we can calculate the relationship between them as follows:


(6) $${\text{W}}_{\mathrm{DD}}\left({\text{d}}_{i},{\text{d}}_{j}\right)=\frac{\sum_{p_{x}\in d_{i}}{\text{W}}_{\mathrm{PD}\left(p_{x},d_{j}\right)}+\sum_{p_{y}\in d_{j}}{\text{W}}_{\mathrm{PD}}\left({\text{p}}_{y},{\text{d}}_{i}\right)}{\left|{\text{d}}_{i}\right|+\left|{\text{d}}_{j}\right|}$$

Obviously, based on above Equation (6), we can easily construct a weighted DDI network *WDIN* = < ***D_DD_***, ***E_DD_*** > as follows: Let ***E_DD_*** denote the set of edges between domains in WDIN, here, for any two given domains ***d_i_*** and **d_*j*_** in ***D_DD_***, if and only if there is ***W_DD_***(***d_i_***,***d_j_***) > 0, we define that there is an edge between them in WDIN, and at the same time, the weight of the edge between ***d_i_*** and **d_*j*_** is ***W_DD_***(***d_i_***, ***d_j_***).

### Construction of the Weighted PDI Network

Based on above Equations (4)–(6), it is obvious that we can construct a new *(***N_P_*** + ***N_D_***) × (***N_D_*** + ***N_P_***)* dimensional matrix ***M_PD_*** as follows:


(7) $${\text{M}}_{\mathrm{PD}}=\left[\begin{array}{cc} {\text{W}}_{\mathrm{PP}} & {\text{W}}_{\mathrm{PD}}\\ {\text{W}}_{\mathrm{PD}}^{T} & {\text{W}}_{\mathrm{DD}} \end{array}\right]$$

Here, ${\text{W}}_{\mathrm{PD}}^{T}$ is a transport matrix of ***W_PD_***. Based on above matrix ***M_PD_***, we can easily construct a novel weighted PDI network *WPDIN* = < ***D_PD_***, ***E_WPD_*** > as follows: Let DPD={pd1,pd,2…,pdNP,pdNP+1,pdNP2,…,pdNP+ND} = {***p_1_***, ***p_2_***, …, **p*_N_P__***, ***d_1_***, ***d_2_***, …, ***d_N_D__***} represent the set of nodes in WPDIN, and ***E_WPD_*** denote the set of edges in WPDIN, then, for any two given nodes ***pd_i_*** and ***pd_j_*** in ***D_PD_***, if and only if there is ***W_PP_***(*pd*_*i*_, *pd*_*j*_) > 0 or ***W_PD_***(***pd_i_***, ***pd_j_***) > 0 or ***W_DD_***(***pd_i_***, ***pd_j_***) > 0, there is an edge between them in ***E_WPD_***, and moreover, the weight of the edge between them is ***M_PD_***(***pd_i_***, ***pd_j_***).

### Calculation of Initial Scores for Proteins

For any given protein node pi in WPDIN, in this section, we will assign an initial score for it based on the functional features extracted from the subcellular localization information of proteins, and the conservative features provided by orthologous information of proteins. Firstly, we will download the orthologous information of proteins from the InParanoid database (Mewes et al., 2006; Gabriel et al., 2010) and the subcellular localization information of proteins from the COMPART-MENTS database (Binder et al., 2014; Min et al., 2017). And then, for convenience, let ***Np***(***i***) represent the total number of proteins relating to the *i-th* subcellular localization, ***N_L_*** denote the total number of different subcellular localizations downloaded above, and ***S***(***p_i_***) represent the set of subcellular locations associating with ***p_i_***. Hence, we can calculate a score for ***p_i_*** based on the subcellular localization information as follows:


(8) $$\text{Subcell}\_\text{Score}\left({\text{p}}_{i}\right)={\text{max}}_{j\in S\left(p_{i}\right)}\text{Subcell}\left(j\right)$$

Where,


(9) $$\text{Subcell}\left(j\right)=\frac{{\text{N}}_{p}\left(j\right)}{{\text{max}}_{1\leq k\leq N_{L}}\left(N_{p}\left(k\right)\right)}$$

Next, let *Hom*(*p*_*i*_) denote the score of *p*_*i*_ in the downloaded homologous information and *N_H_* denote the total number of proteins with homologous information, then, we can calculate another score for *p_i_* based on the homologous information as follows:


(10) $$Hom\_Score\left(p_{i}\right)=\frac{Hom\left(p_{i}\right)}{\max_{1\leq j\leq N_{H}}Hom\left(p_{i}\right)}$$

Finally, through integrating above two kinds of scores together, we can obtain an initial score for *p_i_* as follows:


(11) $$Initial\_Score\left(p_{i}\right)=\frac{Subcell\_Score\left(p_{i}\right)+Hom\_Score\left(p_{i}\right)}{2}$$

### Construction of the Prediction Model KTCSPM

#### Establishment of the Key Target Convergence Sets

**FIGURE 1:**
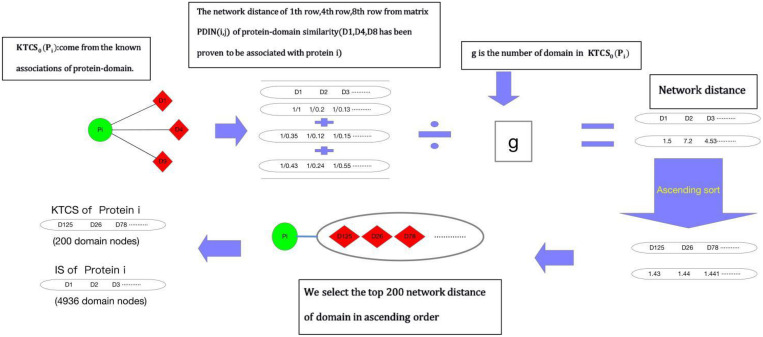
Flow chart of constructing KTCS for any given protein node *p_i_* in WPDIN.

Before implementing random walk with restart on WPDIN, as shown in Figure 1, each node in WPDIN will establish a unique KTCS first according to the following steps:

Step 1: For any given protein node *p_i_* in WPDIN, we define its original KTCS as the set of all domain nodes associating with *p_i_*, that is the original KTCS of *p_i_* is *KTCS*_0_(p_i_) = {*d*_*k*_ *|M_PD_(d*_*k*_, *p_i_*) = 1, *d*_*k*_ ∈ *D*_*DD*_}. Similarly, for any given protein domain node *d_j_*, we can define its original KTCS as *KTCS*_0_(*d_j_*) = {*p_k_|M_PD_(d_j_*, *p_k_*) = 1, *p*_*k*_∈*D*_*PP*_}.

Step 2: For any given protein node *p_i_* in WPDIN, ∀*d*_*k*_ ∈ *KTCS*_0_(*p*_*i*_) *and* ∀*d*_*t*_ ∈ *D*_*DD*_, we define the network distance between *d_k_* and *d_t_* in WPDI as follows:


(12) $$AD\left(d_{k},d_{t}\right)=\frac{1}{W_{DD}\left(d_{k},d_{t}\right)}$$

Similarly, for any given domain node *d_i_* in WPDIN, ∀*p*_*k*_ ∈ *KTCS*_0_(d_i_) *and* ∀*p*_*t*_ ∈ *D*_*PP*_, we can define the network distance between *p_k_* and *p_t_* in WPDI as follows:


(13) $$AD\left(p_{k},p_{t}\right)=\frac{1}{W_{PP}\left(p_{k},p_{t}\right)}$$

Step 3: According to the above Equations (13, 14), for any given protein node *p_i_* or domain node *d_j_* in WPDIN, we define the KTCS (*d_j_*) of *d_j_* as the set of first 200 protein nodes in WPDIN that have the minimum average network distance to nodes in *KTCS*_0_*(d_j_)*, and the *KTCS (p_i_)* of *p_i_* as the set of first 200 domain nodes in WPDIN that have the minimum average network distance to nodes in *KTCS*_0_(*p*_*i*_). Therefore, it easy to know that these 200 protein nodes in *KTCS (d_j_)* may belong to *KTCS*_0_(*d*_*j*_) or may not belong to *KTCS*_0_(*d*_*j*_), and these 200 domain nodes in *KTCS(p_i_)* may belong to *KTCS*_0_(*p*_*i*_) or may not belong to *KTCS*_0_(*p*_*i*_) as well.

#### Random Walk With Restart in WPDIN

**FIGURE 2:**
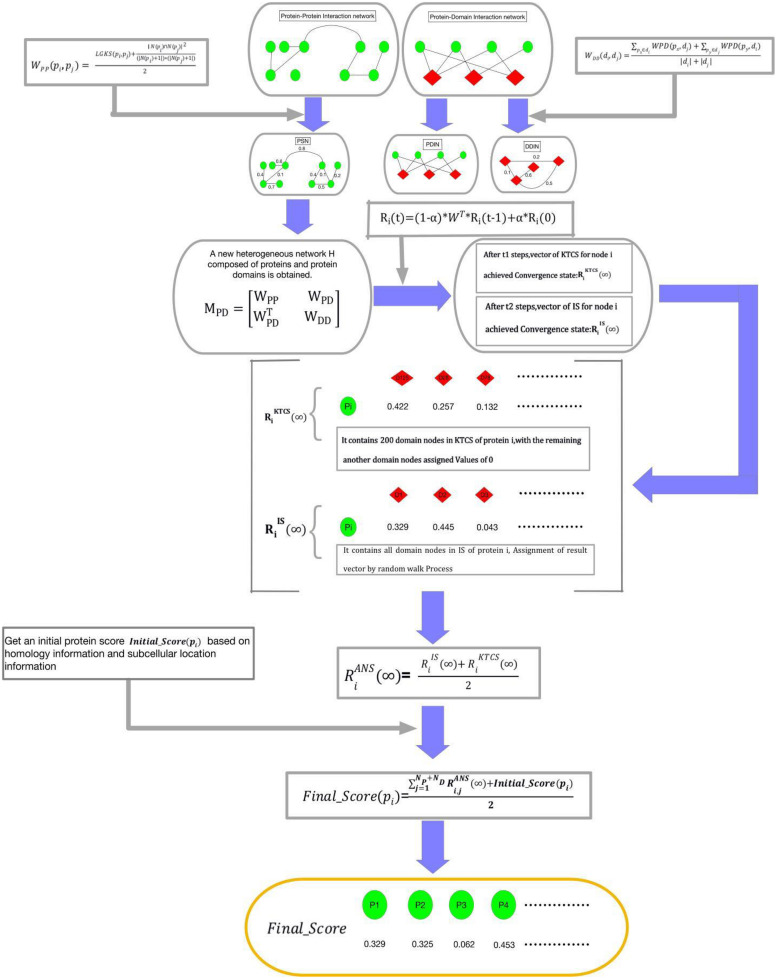
Schematic diagram of the construction process of KTCSPM, where green nodes represent proteins and red nodes represent domains.

The transition process of a walker from a starting node in the network to other nodes with a given probability is called the method of Random walk. Based on the assumption that there is a correlation between essential proteins and domains, as shown in Figure 2, the random walk process of KTCSPM can be mainly divided into the following steps:

Step 1: For a walker, before it starts to walk randomly in WPDIN, we can first obtain a transition probability matrix W for it as follows:


(14) $$W\left(i,j\right)=\frac{M_{PD}\left(i,j\right)}{\sum_{k=1}^{N_{P}+N_{D}}M_{PD}\left(i,k\right)}$$

Step 2: Moreover, for any given node *pd*_*i*_ in WPDIN, we can as well obtain an initial probability vector *R_i_*(0) for the walker as follows:


(15) $$R_{i}\left(0\right)=\left(R_{i,1}\left(0\right),R_{i,2}\left(0\right),\ldots R_{i,j}\left(0\right),\ldots R_{i,N_{P}+N_{D}}\left(0\right)\right)$$

(16) $$R_{i,j}\left(0\right)=W\left(i,j\right),j=1,2,3,\ldots N_{P}+N_{D}$$

Step 3: Next, while starting a walk, the walker will select a node (for convenience, let it be *pd*_0_) in WPDIN randomly as its initial location of this walk, where *pd*_0_ may be a protein node or a domain node. Supposing that after walking *t*-1 hops, the walker reaches the current node *pd*_*i*_ in WPDIN, then, we can further calculate a new walking probability vector *R_i_*(*t*) for it as follow:


(17) $$R_{i}\left(t\right)=\left(1-\alpha \right)*W^{T}*R_{i}\left(t-1\right)+\alpha *R_{i}\left(0\right)$$

Here, α(*0 <* α < 1) is a parameter for adjusting weights between *R_i_*(0) and *R_i_*(*t*-1). Moreover, for convenience, let *R_i_*(*t*) = (*R*_*i*,1_(*t*), *R*_*i*,2_(*t*), …, *R*_*i*,*j*_(*t*), …, *R*_*i*,*N*_*P*_*N*_*D*__(*t*))^*T*^, where *R*_*i*,*j*_(*t*) denotes the walking probability that the walker will walk from its current location *pd*_*i*_ to the node *pd*_*j*_ at its *t-th* hop. Here, it is worth noting that for the starting node *pd*_0_, since the walker can be considered to reach pd_0_ from *pd*_0_ after zero hops, therefore, for the starting node *pd*_0_, the walker can obtain an initial probability vector *R_0_*(0), and a walking probability vector *R_0_*(1).

Step 4: Assuming that the walker has walked from a node *pd*_*i*_ to a current node *pd*_*j*_ after *t*-1 hops during its random walk in WPDIN, the walk probability vectors calculated by the walker at *pd*_*i*_ and *pd*_*j*_ are *R_i_*(*t*-1) and *R_j_*(*t*), respectively, if the L1 norm between *R_i_*(*t*-1) and *R_j_*(*t*) satisfies ||*R*_*j*_(*t*)−*R*_*i*_(*t*−1)||_1_ ≤ 10^−6^, then we define that the walking probability vector *R_j_*(*t*) has reached a stable state at its current location. Moreover, after the walker having obtained a stable walking probability at each node in WPDIN, for convenience, we will define the stable probability obtained by the walker at any given node *pd*_*k*_ in WPDIN as *R_k_*(∞), and then, we can construct a stable walking probability matrix *K* (∞) as follows:


(18) $$K\left(\infty \right)=\left[\begin{array}{cc} K_{1} & K_{2}\\ K_{3} & K_{4} \end{array}\right]={\left(R_{1}\left(\infty \right),R_{2}\left(\infty \right),R_{3}\left(\infty \right).....,R_{N_{P}N_{D}}\left(\infty \right)\right)}^{T}$$

where, *K*_1_ is a *N*_*P*_ × *N*_*P*_ dimensional matrix, *K*_2_ is a *N*_*P*_ × *N*_*D*_ dimensional matrix, *K*_3_ is a *N*_*D*_ × *N*_*P*_ dimensional matrix, and *K*_4_ is a *N*_*D*_ × *N*_*D*_ dimensional matrix. Thereafter, it is obvious that *K*_2_ and *K*_3_ will be the final result matrices, which can be adopted to predict potential essential proteins.

According to above steps of KTCSPM, it is easy to see that, for any node *pd*_*i*_ in WPDIN, a stable walking probability vector *R*_*i*_(∞) = (*R*_*i*,1_(∞),*R*_*i*,2_(∞), …, *R*_*i*,*j*_(∞), …, *R*_*i*,*N*_*P*_ + *N*_*D*__(∞))^*T*^ will be obtained by the walker. For convenience, we denote the node set DPD in WPDIN as the IS. Therefore, we can redefine the stable probability R_*i*_(∞) as RISi(∞). However, through observing RISi(∞), it is easy to find that the walker will stop its random walking only after the walking probability vector calculated at each node in WPDIN is stable. In the face of large data, this mechanism is obviously very time-consuming. Hence, in order to speed up the convergence speed of KTCSPM and reduce the experimental execution time, based on the concept of KTCS defined above, when constructing the vector *R_i_*(*t*) = (*R*_*i*,1_(*t*),*R*_*i*,2_(*t*), …, *R*_*i*,*j*_(*t*), …, *R*_*i*,*N*_*P*_ + *N*_*D*__(*t*))^*T*^ at the node *pd*_*i*_, if the *j-th* node *pd*_*j*_ ∈ *KTCS (pd*_*i*_*)* in WPIND, then *R*_*i*,*j*_(*t*) will be remained unchanged, otherwise we will redefine *R*_*i*,*j*_(*t*) = *0*. Thus, the walking probability vector at *pd*_*i*_ will be changed to RiKTCS(t) and the stable walking probability at *pd*_*i*_ will be changed to RiKTCS(∞). Obviously, the stable state of RiKTCS(∞) can be achieved faster than that of RiIS(∞). However, considering that there may be some nodes not belonging to *KTCS* (*pd*_*i*_) but relating to the target, therefore, in order to avoid any omissions, at any given node *pd*_*i*_ in WPIND, we will construct a novel final stable walking probability vector $R_{i}^{ANS}\left(\infty \right)={\left(R_{i,1}^{ANS}\left(\infty \right),R_{i,2}^{ANS}\left(\infty \right),\ldots ,R_{i,j}^{ANS}\left(\infty \right),\ldots ,R_{i,N_{P}+N_{D}}^{ANS}\left(\infty \right)\right)}^{T}$ by combining RiIS(∞) with RiKTCS(∞) as follows:


(19) $$R_{i}^{ANS}\left(\infty \right)=\frac{R_{i}^{IS}\left(\infty \right)+R_{i}^{KTCS}\left(\infty \right)}{2}$$

Step 5: For any protein node *p_i_* in WPIND, according to the final stable walking probability vector $R_{i}^{ANS}\left(\infty \right)$ and the initial protein score *Initial_Score* (*p_i_*) obtained above, it is obvious that a novel final feature score *Final_Score* (*p_i_*) can be calculated as follows:


(20) $$Final\_Score\left(p_{i}\right)=\frac{\sum_{j=1}^{N_{P}+N_{D}}R_{i,j}^{ANS}\left(\infty \right)+Initial\_Score\left(p_{i}\right)}{2}$$

#### Algorithm KTCSPM

##### Input

Original PPI network, original protein-domain network, domain data, subcellular data, orthologous data, and the proportion regulation parameters α.

##### Output

Proteins final score *Final*_*Score*(*p*_*i*_).

Step 1: Establishing the heterogeneous network according to formulas (1–7);

Step 2: Calculating proteins initial score by orthologous data and subcellular data according to formulas (8–11);

Step 3: Establishing the KTCS according to formulas (12, 13);

Step 4: Establishing the transition probability matrix W according to formula (14);

Step 5: Calculating a stable walking probability vector R_i_(t) according to formulas (15–17);

Step 6: Establishing stable walking probability matrix K (∞) according to formula (18); and

Step 7: Outputting the final score of protein according to formula (19);

## Results

### Experimental Data

In this section, extensive experiments will be done to compare KTCSPM with representative methods. And during experiments, the domain data is downloaded from the Pfam database (Bateman et al., 2014). The subcellular location data is derived from the COMPARTMENTS database (Binder et al., 2014), in which, the following classifications of the subcellular interstitium related to the basic proteins of eukaryotic cells are included: Golgi bodies, endoplasm, cytoplasm, cytoskeleton, vacuoles, endosomes, mitochondria, plasma, peroxomes, and nuclei, etc. Besides, the reference bases of the essential genes of Scerevisiae are collected from MIPS (Mewes et al., 2006), SGD (Cherry et al., 1998), DEG (Zhang and Lin, 2009), and SGDP (Saccharomyces Genome Deletion Project, 2012). In the dataset downloaded from the DIP database, there are 5,093 proteins in total, in which, 1,167 are essential and 3,526 are non-essential. In the dataset downloaded from the GAVIN database, there are a total of 1,855 proteins, in which, 714 are essential proteins.

### Comparison Between KTCSPM and Competitive Methods

**FIGURE 3:**
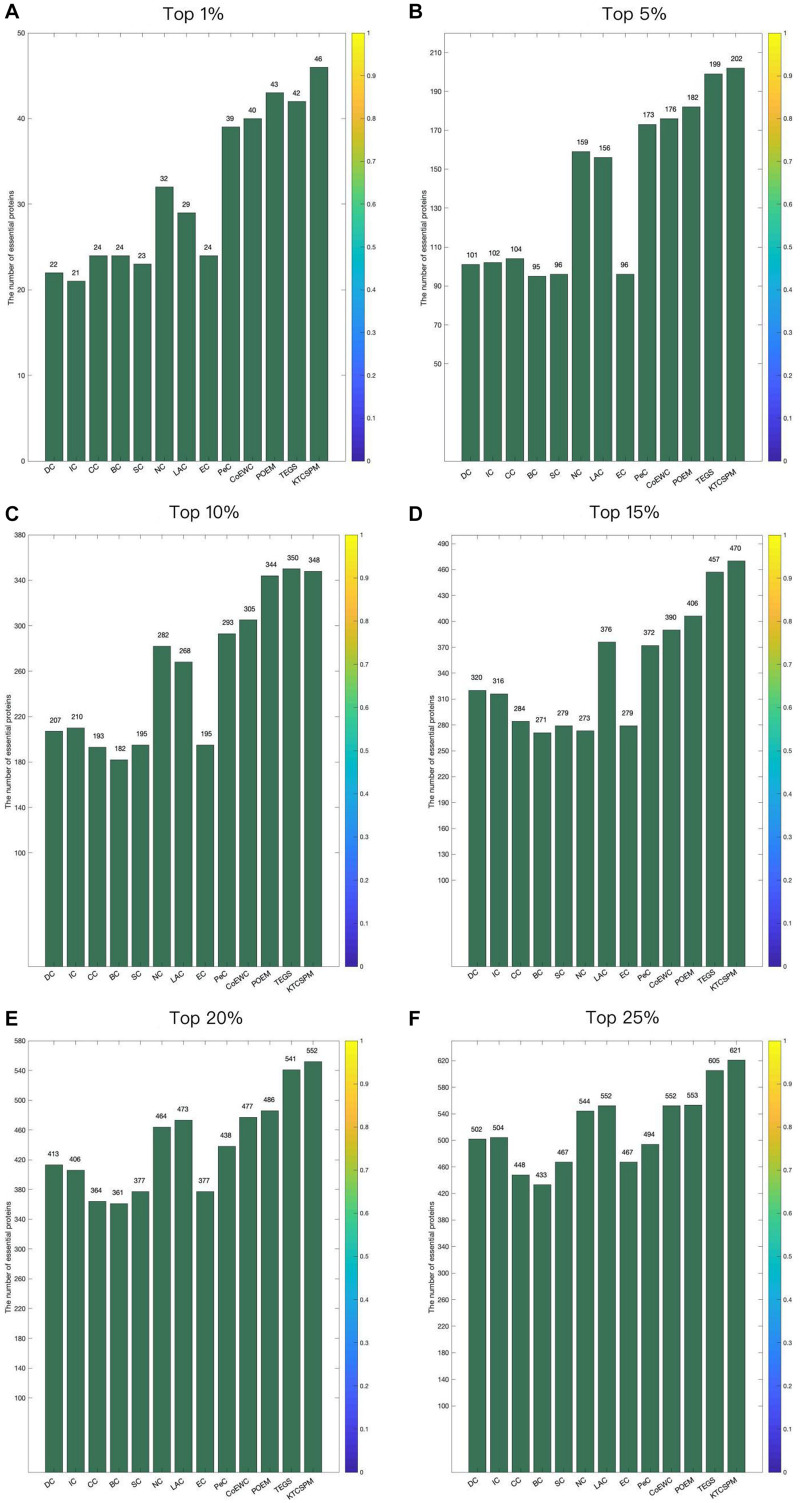
**(A)** Top 1% ranked proteins. **(B)** Top 5% ranked proteins. **(C)** Top 10% ranked proteins. **(D)** Top 15% ranked proteins. **(E)** Top 20% ranked proteins. **(F)** Top 25% ranked proteins. In this Figure shows the predictive accuracy between KTCSPM and 12 competitive methods including IC, CC, BC, SC, NC, LAC, EC, PeC, CoEWC, POEM, and TEGS.

In order to verify the predictive performance of KTCSPM, in this section, we will compare it with several representative methods such as DC (2001), IC (1989), And So-called Centrality (CC) (2014), Bee-tweenness Centrality (BC) (2005), SC (2003), NC (2005), PeC (2012),LAC (2011), CoEWC (2014), POEM (2017), and TEGS (2019) based on the DIP database and the Gavin database separately. Figure 3 shows the comparison results between KTCSPM and these competitive methods. From observing Figure 3, it is obvious that the prediction accuracy of KTCSPM is significantly better than that of all these competing methods in from top 1 to 25% predicted essential proteins. In particular, KTCSPM can achieve a reliable prediction accuracy rate of 90.21% in the top 1% ranked key proteins.

### Validation With Jackknife Methodology

**FIGURE 4:**
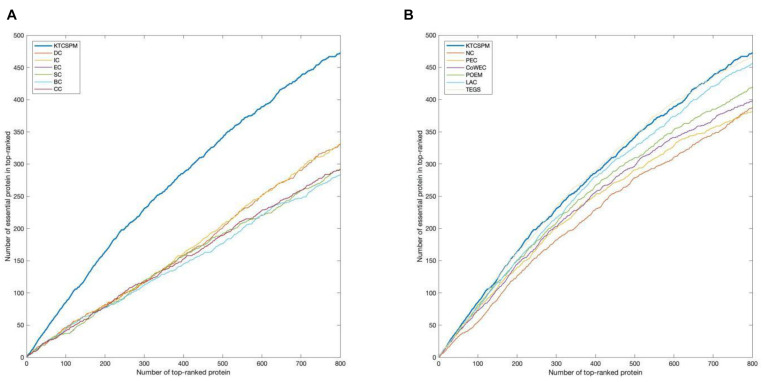
The comparison results between key target convergence sets based prediction model (KTCSPM) and 12 competitive methods based on the DIP database under the jackknife methodology. **(A)** Comparison results between KTCSPM and DC, IC, EC, SC, BC, and CC. **(B)** Comparison results between KTCSPM and NC, PeC, CoWEC, POEM, LAC, and TEGS.

For a comprehensive and accurate comparison, in this section, we will adopt the Jackknife methodology (Holman et al., 2009) to compare the predictive performances between KTCSPM and above mentioned competing methods. Experimental results are shown in Figure 4, from which, it can be clearly seen that the jackknife curve of KTCSPM is higher than that of all these state-of-the-art predictive methods. Although in Figure 4B, the jackknife curves of KTCSPM and TEGS have multiple intersections, however, when the number of ranked proteins is bigger than 600, the predictive results of KTCSPM will become continuously higher than that of TEGS. Therefore, according to both Figures 4A,B, we can draw a conclusion that KTCSPM can achieve better predictive performance than all these representative methods in predicting essential proteins.

### Validation by Precision-Recall Curves and ROC Curves

**FIGURE 5:**
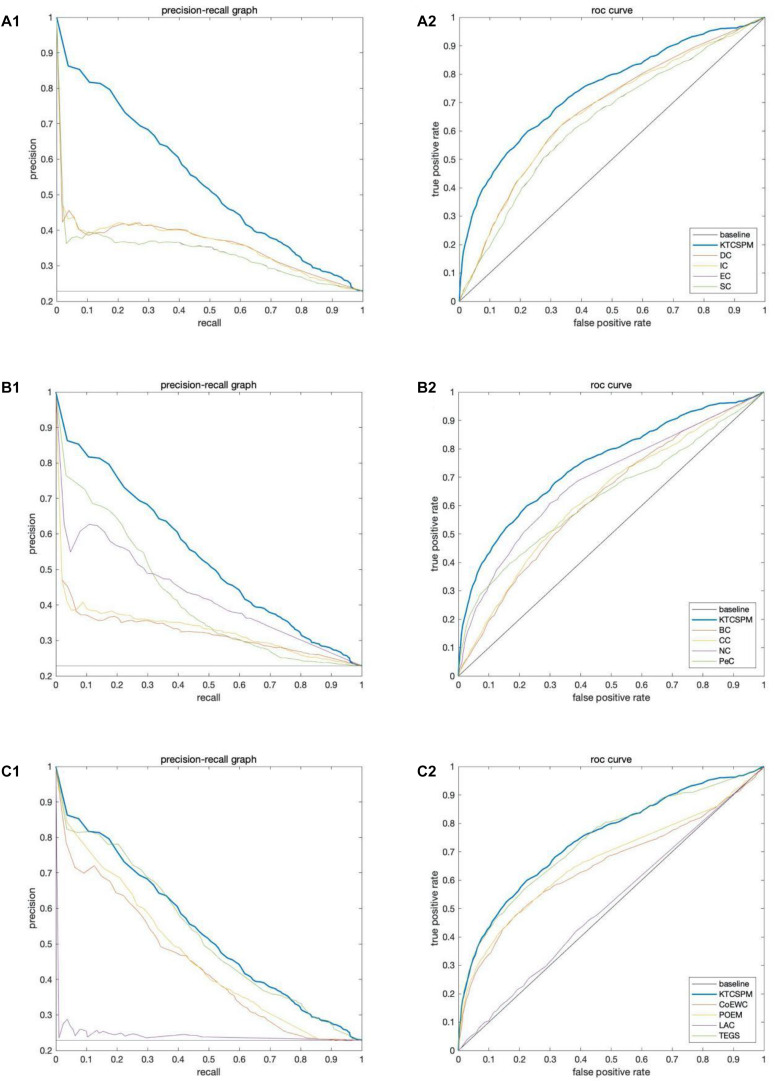
Comparisons of performance between key target convergence sets based prediction model (KTCSPM) and 12 competitive models under the PR curve and ROC curve based on the DIP database. Panels **(A1–C1)** are comparison results of PR curves between KTCSPM and 12 competitive models. Panels **(A2–C2)** are comparison results of ROC curves between KTCSPM and 12 competitive models.

In this section, ROC curve (receiver operating characteristic) and precision-recall curves (PR) will be adopted to measure the performance of KTCSPM. Researches show that the larger the area under the ROC curve (AUC), the better the model performance, and in addition, when AUC = 0.5, the model performance will be in a random state. Moreover, when PR curves are adopted to evaluate predictive models, more comprehensive feedbacks on performances of predictive models can be obtained by using different validation methods. And as a result, Figure 5 shows the comparisons of performance between KTCSPM and 12 competitive prediction models under the PR curve and ROC curve separately. From the Figures 5A1,2,B1,2, it can be seen that when KTCSPM is compared with SC, EC, DC, IC, BC, CC, NC, PeC, the area under the PR curve (AUC), and ROC curve display results show that KTCSPM is superior. For these methods, by observing a3 and b3, it can be seen that when KTCSPM is compared with TEGS and POEM methods, the gap becomes smaller and there is overlap, but even so, the prediction performance of KTCSPM is still better than the 12 methods.

### Analysis of the Differences Between KTCSPM and Competitive Methods

**FIGURE 6:**
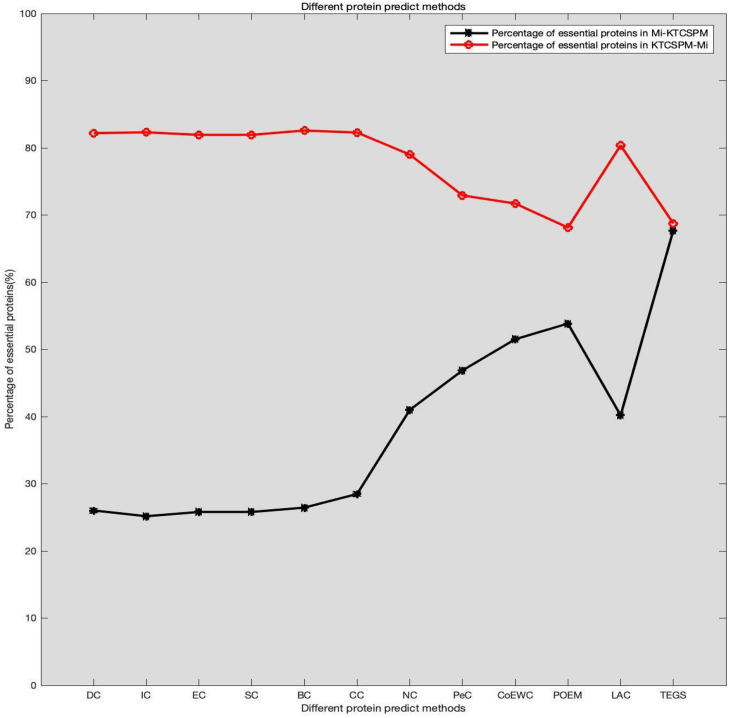
Comparison results between KTCSPM and 12 competing methods, where the *X* axis denotes the competing methods including DC, IC, EC, SC, BC, CC, NC, PEC, COEWC, POEM, LAC, TEGS, and the *Y* axis represents the proportion of true essential proteins predicted by each method.

It can be seen from above descriptions that KTCSPM can achieve satisfactory predictive effects. In this section, we will further analyze the differences between KTCSPM and 12 competing methods by calculating the number of overlaps of first 200 predicted proteins. comparison results are shown in Figure 6, where Mi represents one of these 12 competitive methods, | KTCSPM-Mi| denotes the number of proteins detected by KTCSPM but not by Mi, | Mi-KTCSPM| means the number of proteins detected by Mi but not by KTCSPM. Obviously, according to the curve trends in Figure 6, we can see that the ratio of essential proteins predicted by KTCSPM is much higher than that predicted by anyone of these 12 competing methods, which means that KTCSPM can screen out more essential proteins not found by Mi, and demonstrates that KTCSPM can achieve much better predictive performance as well.

### Prediction Performance of KTCSPM Based on the Gavin Database

In this section, in order to further verify the adaptability of KTCSPM, we will further compare it with 11 competitive methods based on the Gavin database, and comparison results are shown in the following Table 1.

**TABLE 1:** Comparison results between KTCSPM and 11 state-of-the-art methods including DC, IC, CC, BC, NC, EC, PeC, CoEWC, ION, and POEM based on the Gavin database, where the Gavin database consists of 1,855 essential proteins.

Method	1%(19)	5%(93)	10%(196)	15%(279)	20%(371)	25%(464)
DC	7	36	101	158	222	264
IC	16	55	119	163	213	254
CC	11	45	93	135	180	221
BC	9	40	85	122	162	201
SC	9	36	87	130	190	240
NC	11	51	123	170	213	259
EC	0	38	94	134	166	209
PeC	15	69	142	193	238	285
CoEWC	16	69	136	190	237	275
ION	17	73	150	207	263	312
POEM	17	74	148	199	249	296
KTCSPM	17	75	160	216	269	315

### Effects of Parameters on Performance of KTCSPM

In this section, we will estimate the effects of parameters on the prediction performance of KTCSPM. First, as for the parameter γ_*p*_ in Equation (1), we will set its value to one based on precedents (Twan et al., 2011). However, as for the parameter in Equation (17), as illustrated in Table 2, we will set its value from 0.1 to 0.9, and evaluate its impacts on the prediction performance of KTCSPM. Through observing Table 2, it is easy to see that when is set to 0.3, KTCSPM can achieve the best prediction effect. Moreover, it can be clearly seen that KTCSPM remains robust to different values of, which means that KTCSPM is not sensitive to the values of α.

**TABLE 2:** Influence of the parameter α on prediction accuracy of KTCSPM based on the DIP database.

Rank α	0.1	0.2	0.3	0.4	0.5	0.6	0.7	0.8	0.9
Top 1%	46	46	46	46	46	45	45	44	45
Top 5%	200	200	202	201	200	199	202	200	202
Top 10%	347	247	348	346	340	347	348	350	348
Top 15%	468	468	470	466	468	465	460	467	467
Top 20%	550	550	552	549	545	550	552	551	550
Top 25%	618	620	621	620	619	619	620	619	621

## Discussion

It is time consuming and energy consuming to predict essential proteins through traditional biological experiments, so it has become a hot topic in the field of bioinformatics to build mathematical models to predict essential proteins. In this manuscript, a new prediction model called KTCSPM is proposed, in which, a weighted PDI network constructed by integrating a weighted PPI network and a weighted DDI network first, and then, based on the concepts of KCS and IS, a predictive method is further designed to infer potential key proteins in the weighted PDI network based on the random walk with restart. Finally, extensive experiments have demonstrated the predictive superiority of KTCSPM. At present, some methods have been proposed to infer potential disease related miRNAs such as RWRMDA (Chen et al., 2012), RLSMDA (Chen and Yan, 2014) and RBMMMDA (Chen et al., 2015), in the future, KTCSPM may also be applied to predict potential associations between miRNAs, and diseases.

## Conclusion

In this manuscript, the main contributions are as follows: (1) A novel weighted PDI network is designed by combining a weighted PPI network with a weighted DDI network. (2) The concept of network distance is introduced, and the KTCS and the IS are established for nodes in the weighted PDI network. (3) Based on the concepts of KTCS and IS, an improved random walk with restart algorithm is proposed to recognize essential proteins. By comparing with existing state-of-the-art predictive models, it is proved that KTCSPM can achieve better predictive performance in detecting essential proteins.

## Data Availability Statement

The datasets presented in this study can be found in online repositories. The names of the repository/repositories and accession number(s) can be found in the article/Supplementary Material.

## Author Contributions

JP conceived this research. JP, LW, and LK were improved on the basis of the original design model. JP and ZZ wrote the manuscript. YT and ZC provided advice and supervision on the research. All authors contributed to the article and approved the submitted version.

## Conflict of Interest

The authors declare that the research was conducted in the absence of any commercial or financial relationships that could be construed as a potential conflict of interest.

## Publisher’s Note

All claims expressed in this article are solely those of the authors and do not necessarily represent those of their affiliated organizations, or those of the publisher, the editors and the reviewers. Any product that may be evaluated in this article, or claim that may be made by its manufacturer, is not guaranteed or endorsed by the publisher.
